# Retraction: Green fabricated zinc oxide nanoformulated media enhanced callus induction and regeneration dynamics of *Panicum virgatum* L.

**DOI:** 10.1371/journal.pone.0345380

**Published:** 2026-03-23

**Authors:** The PLOS One Editors

The *PLOS One* Editors retract this article [1] because it was identified as one of a series of submissions for which we have concerns about manipulation of the publication process. We regret that the issues were not addressed prior to the article’s publication.

NJ and KSA did not agree with the retraction. SS, SI, SAnwaar, NA, SAlam, MI, TFK, and SZH either did not respond directly or could not be reached.
